# Integrating population genetics to define conservation units from the core to the edge of *Rhinolophus ferrumequinum* western range

**DOI:** 10.1002/ece3.5714

**Published:** 2019-10-12

**Authors:** Orianne Tournayre, Jean‐Baptiste Pons, Maxime Leuchtmann, Raphael Leblois, Sylvain Piry, Ondine Filippi‐Codaccioni, Anne Loiseau, Jeanne Duhayer, Inazio Garin, Fiona Mathews, Sébastien Puechmaille, Nathalie Charbonnel, Dominique Pontier

**Affiliations:** ^1^ CBGP INRA CIRAD IRD Montpellier SupAgro Université de Montpellier Montferrier‐sur‐Lez Cedex France; ^2^ LabEx ECOFECT «Ecoevolutionary Dynamics of Infectious Diseases» Université de Lyon Lyon France; ^3^ Nature Environnement Surgères France; ^4^ Department of Zoology and Animal Cell Biology University of the Basque Country Leioa The Basque Country; ^5^ College of Life Sciences University of Sussex Falmer UK; ^6^ ISEM Univ Montpellier CNRS EPHE IRD Montpellier France; ^7^ Groupe Chiroptères de Midi‐Pyrénées (CREN‐GCMP) Toulouse France; ^8^ CNRS Laboratoire de Biométrie et Biologie Évolutive UMR5558 Université Lyon 1 Université de Lyon Villeurbanne France

**Keywords:** Chiroptera, connectivity, conservation, demographic inference, microsatellites, population genetics

## Abstract

The greater horseshoe bat (*Rhinolophus ferrumequinum*) is among the most widespread bat species in Europe but it has experienced severe declines, especially in Northern Europe. This species is listed Near Threatened in the European *IUCN Red List of Threatened Animals*, and it is considered to be highly sensitive to human activities and particularly to habitat fragmentation. Therefore, understanding the population boundaries and demographic history of populations of this species is of primary importance to assess relevant conservation strategies. In this study, we used 17 microsatellite markers to assess the genetic diversity, the genetic structure, and the demographic history of *R. ferrumequinum* colonies in the western part of its distribution. We identified one large population showing high levels of genetic diversity and large population size. Lower estimates were found in England and northern France. Analyses of clustering and isolation by distance suggested that the Channel and the Mediterranean seas could impede *R. ferrumequinum* gene flow. These results provide important information to improve the delineation of *R. ferrumequinum* management units. We suggest that a large management unit corresponding to the population ranging from Spanish Basque Country to northern France must be considered. Particular attention should be given to mating territories as they seem to play a key role in maintaining high levels of genetic mixing between colonies. Smaller management units corresponding to English and northern France colonies must also be implemented. These insular or peripheral colonies could be at higher risk of extinction in the near future.

## Graphical abstract

In this study, we used 17 microsatellite markers to assess the genetic diversity, the genetic structure, and the demographic history of the greater horseshoe bat (*Rhinolophus ferrumequinum*) colonies in the Western European part of its distribution. We found high levels of genetic diversity and large population size in the European mainland and lower estimates in England and northern France. We suggest that a large management unit corresponding to the European mainland population must be considered. Smaller management units corresponding to English and northern France colonies must also be implemented because they could be at higher risk of extinction in the near future.

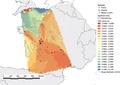

## 1 INTRODUCTION

Biodiversity is dramatically declining at an accelerating rate for most animal groups (Butchart et al., 2010; Hoffmann et al., 2010; Sánchez‐Bayo & Wyckhuys, 2019). According to the IUCN Red List, more than 26,500 species (27% of all assessed species) are threatened with extinction. This phenomenon results from a combination of ecological factors (e.g., habitat fragmentation and destruction, pollution, introduction of invasive species, and climate change) that affect population sizes and connectivity. As a consequence, these populations become strongly exposed to the negative impacts of inbreeding and genetic drift (Frankham, 2005). Preserving the genetic diversity of such small and isolated populations is therefore essential to avoid inbreeding depression, to maintain genetic variability that may be useful for adaptation, in particular in response to environmental changes, and ultimately, to promote population persistence (Reed & Frankam, 2003). To address this issue and before drawing efficient conservation programs, an important prerequisite is to gather knowledge on population boundaries and demography. In recent decades, population genetics has been combined with more classical ecological studies to infer population demographic features, including the detection of recent demographic declines or the quantification of connectivity between populations (e.g., Vignaud et al., 2014; Vonhof & Russell, 2015). Ultimately, these population genetics studies may help delineating functional and evolutionary conservation units such as “Management Units,” which are appropriate for species monitoring and management. There are several ways to define management units, among which are the assessment of panmixia (Moritz, 1994) or the estimation of population genetic divergence (Palsbøll, Bérubé, & Allendorf, 2007). Designing appropriate management units is thus far from being trivial. There is no general framework for determining at which dispersal rate populations become demographically correlated (therefore requiring a single management unit), and there is no easy way to translate gene flow estimates provided by population genetics into dispersal rates. Yet, too large units may increase the risk of extinction of “cryptic” populations that would require specific strategies. On the other hand, splitting a large population into different conservation units with different strategies may lead to excessive management strategies beyond requirements, or to inappropriate strategies limiting connectivity.

The application of population genetics to such issues of conservation biology has been especially important for endangered species that are difficult to monitor with ecological methods, such as bats. Indeed, bats are very sensitive to climate change and human activities (Jones, Jacobs, Kunz, Willig, & Racey, 2009; Voigt & Kingston, 2016). Almost one‐quarter of bat species in the world are considered to be Threatened and another quarter as Near Threatened (Mickleburgh, Hutson, & Racey, 2002). Temperate‐zone bats are nocturnal, small, highly mobile, and the location of their roosts are often poorly known, characteristics that make monitoring and assessment of their extinction risk difficult (O'Shea, Bogan, & Ellison, 2003). Conservation programs are often established considering local and national scales. Unfortunately, these scales are usually not defined on the basis of biological knowledge on population delineation and demography, but instead conform to administrative borders that rarely correspond to natural ecological boundaries. This is likely to limit the efficiency and coherence of conservation strategies. Population genetics might therefore improve the definition of appropriate management units of bat populations (e.g., Dool, O'Donnell, Monks, Puechmaille, & Kerth, 2016; Ibouroi et al., 2018). Besides, demographic inferences based on population genetics may be particularly relevant to highlight the need of conservation management for bat species. As such, Durrant, Beebee, Greenaway, and Hill (2009) have been able to evidence a recent decline and high levels of inbreeding in British populations of Bechstein's bat (*Myotis bechsteinii*).

Among European bat species, the greater horseshoe bat (*Rhinolophus ferrumequinum*) is particularly relevant to address conservation issues from population genetics. This insectivorous species—which seasonally uses hibernation and maternity roosts—has experienced dramatic declines, particularly in Northern Europe (e.g., Belgium, Luxembourg, England) where it is now considered rare or extinct (Kervyn, Lamotte, Nyssen, & Verschuren, 2009; Mathews et al., 2018; Pir, 2009). In some countries, such as the UK, there is evidence of recent population increases (Mathews et al., 2018). The species is included in Appendix II of Bern Convention, Appendix II of the Bonn Convention, Annex II and Annex IV of the European Directive on the conservation of Natural Habitat and of Wild Fauna and Flora, and is listed in the *IUCN Red List of Threatened Animals* (International Union for the Conservation of Nature, 2017). The reasons for the disappearance of the populations of *R. ferrumequinum* are difficult to identify but it is likely that anthropogenic factors (e.g., intensification of agriculture, urbanization, and loss of roosts) are responsible for it (Froidevaux, Boughey, Barlow, & Jones, 2017; Mathews et al., 2018). It is therefore important to implement conservation programs, at adequate geographical scales and based on a solid knowledge of *R. ferrumequinum* population dynamics.

Previous phylogeographic studies of *R. ferrumequinum* have revealed a unique genetic cluster in Western Europe mainland, ranging from Portugal to Italy (Switzerland apart), that resulted from the expansion of a single population originating from a Western Asian refugium (Flanders et al., 2009; Rossiter, Benda, Dietz, Zhang, & Jones, 2007). However, these studies are based on sparse sampling, especially in mainland Europe (e.g., one location in France). Yet, where populations have been intensively sampled, a strong genetic differentiation was observed at smaller spatial scales (over tens to several hundreds of kilometers), for instance, within the United Kingdom (Rossiter, Jones, Ransome, & Barrattt, 2000). These patterns underline the importance of sampling density in population genetics studies to detect finer genetic clustering and particular population functioning (e.g., source–sink dynamics), despite an apparent lack of genetic differentiation detected over thousands of kilometers. More specifically, only one French location of *R. ferrumequinum* had been included in these previous phylogeographic studies (Flanders et al., 2009; Rossiter et al., 2007, 2000). However, *R. ferrumequinum* distribution in France is very disparate, and its status can be very contrasted between regions (Vincent & Bat Group SFEPM, 2014). These patterns are suggestive of differences in population size, connectivity levels, and therefore extinction risk. Most of the known roosts of *R. ferrumequinum* in France are located on the Atlantic coast (Vincent & Bat Group SFEPM, 2014). The 4th largest hibernating population (about 7,000 individuals) and the 10th largest summer population (about 2,000 individuals) are found in the Poitou‐Charentes region. This region has therefore a strong conservation responsibility to preserve this species, and several programs have been dedicated to study *R. ferrumequinum* at this regional scale. *R. ferrumequinum* population dynamics are being assessed using counts of individuals performed in hibernation and summer roosts by local NGOs for over three decades. *R. ferrumequinum* experiences important seasonal fluctuations in abundance that reflect seasonal changes of roosts. These fluctuations suggest large movements and potential gene flow of bats during winter and summer. However, we still have only limited knowledge about connectivity and genetic mixing between French *R. ferrumequinum* colonies.

In this study, we proposed to identify population boundaries of *R. ferrumequinum* in its western distribution and to infer the demographic history of these populations. More specifically, we aimed at assessing the biological relevance of considering the French Poitou‐Charentes region as a singular Management Unit (MU), as it is currently done on the basis of political constraints. We combined several population genetics approaches based on a dense sampling of *R. ferrumequinum* maternity colonies in the Poitou‐Charentes region and we also added further samples in France, encompassing the northern edge of *R. ferrumequinum* distribution, Spanish Basque Country, England, and Tunisia. This sampling scheme, based on concentric circles of sampling, should be able to identify at which geographical scales *R. ferrumequinum* gene flow may decrease with distance. It should also allow testing for gene flow disruption that might result from population divergence associated with geographic elements such as seas or mountains. We estimated the genetic diversity and genetic effective size of colonies. These are important criteria to assess population viability, so that they may be useful to infer conservation priority. We expected lower levels of genetic diversity and higher levels of genetic differentiation at the edge of *R. ferrumequinum* distribution range. Indeed, peripheral populations are likely to suffer from reduced gene flow, genetic drift, and small effective population size, compared to central populations (central‐margin hypothesis, Eckert, Samis, & Lougheed, 2008). In addition, we examined patterns of genetic differentiation to evaluate the connectivity between *R. ferrumequinum* colonies. We expected a disruption of gene flow between colonies located on either side of the Mediterranean Sea or Channel Sea, as it has already been shown that sea is a barrier to dispersal for several bat species (Castella et al., 2000; García‐Mudarra, Ibáñez, & Juste, 2009). Considering previous studies, we did not expect a strong disruption of gene flow due to the Pyrenees between French and Spanish Basque colonies (Rossiter et al., 2007).

Altogether, our results provide important information about *R. ferrumequinum* population genetics that will complement ecological knowledge gathered by local NGOs to define appropriate management units. In this study, we assumed an intermediate MU definition between those of Moritz (1994) and Palsbøll et al. (2007). Both academic and nonacademic partners were involved in this work to guarantee that the results would directly inform conservation and management action (Britt, Haworth, Johnson, Martchenko, & Shafer, 2018). More broadly, this study illustrates how population genetics may bring important information to delineate bat management units and to design conservation programs of bat species at relevant geographical scales.

## 2 MATERIAL AND METHODS

### 2.1 Ethical statements

Authorization for bat capture in France was provided by the Ministry of Ecology, Environment and Sustainable development over the period 2015–2020 (approval no. C692660703 from the Departmental Direction of Population Protection (DDPP), Rhone, France). All methods were approved by the Museum National d'Histoire Naturelle (MNHN) and the Société Française pour l'Étude et la Protection des Mammifères (SFEPM). Authorization for bat captures in Spanish Basque Country was provided by the corresponding regional Ministries and Councils. Capture and handling protocols followed published guidelines for treatment of animals in research and teaching (Buchanan et al., 2012) and were approved by the Ethics Committee at the University of the Basque Country (Ref. CEBA/219/2012/GARIN ATORRASAGASTI). Authorization for capture in England was given by Natural England (Ref. 2017‐29766‐SCI‐SCI) and sampling was undertaken under license from the Home Office (PPL 3003431).

### 2.2 Biological material

**Figure 1:**
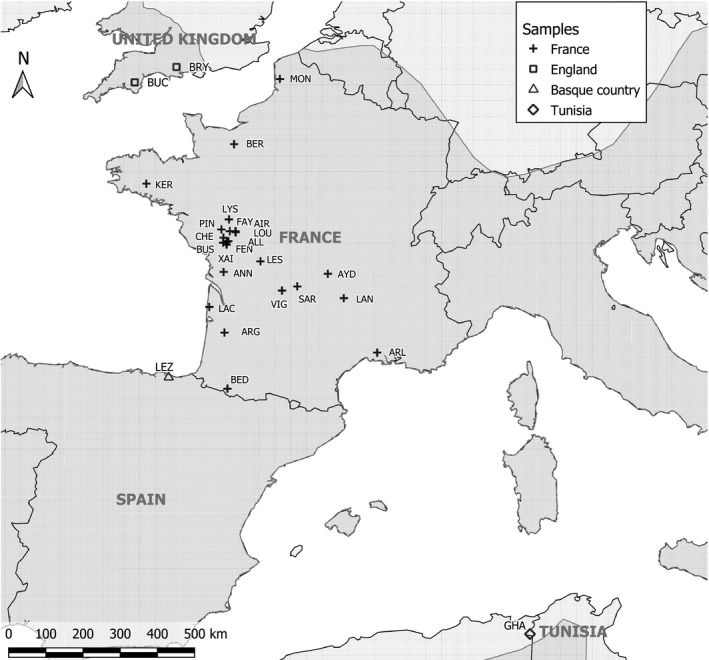
Map of the colonies included in this study. The geographic range of the species is colored in gray according to the IUCN data (2016). Bryanston (BRY), Buckfastleigh (BUC), Montreuil‐sur‐mer (MON), Kernascleden (KER), Lys‐Haut‐Layon (LYS), Allonne (ALL), Le Busseau (BUS), Le Pin (PIN), La Chapelle‐Saint‐Etienne (CHE), Xaintray (XAI), Airvault (AIR), Saint‐Loup‐sur‐Thouet (LOU), Faye l'Abesse (FAY), Fenioux (FEN), Lessac (LES), Annepont (ANN), Sarran (SAR), Vignols (VIG), Aydat (AYD), Langeac (LAN), Lacanau (LAC), Argelouse (ARG), Arles (ARL), Bedous (BED), Lezate (LEZ), and El Feidja National Park (GHA)

This study is based on 950 captured individuals: 864 were captured in 23 maternity colonies in France (summer 2016 to 2018), 28 were captured in one maternity colony in the Spanish Basque Country (summer 2012), 36 were captured in two maternity colonies in England (summer 2018), and 22 were captured in one maternity colony in Tunisia (Puechmaille, Hizem, Allegrini, & Abiadh, 2012). Details are provided in Table 1 and Figure 1. Maternity colonies correspond to the roosts where female bats gather from May to August, give birth, and rear their young (Ransome & Hutson, 2000). This sampling leads to unbalanced sex and age ratios, with 887 females, 62 males, and one undetermined, and 808 adults, 130 juveniles (less than two years old), and 12 undetermined, respectively. Distances between sampling colonies varied from 2.53 km (closest colonies from western France) to 1,830 km (colonies from England and Tunisia).

**Table 1:** Genetic diversity parameters for each locality studied in France, Spanish Basque Country, England, and Tunisia

Region	Locality code	Locality	*N*	*A*_r_	*pA*	*H*_e_	*F* _is_
France							
Poitou‐Charentes region	AIR	Airvault	31	6.46	0.02	0.74	0.036
	ALL	Allonne	31	6.67	0.03	0.76	0.008
	ANN	Annepont	23	6.42	0.1	0.72	0.027
	BUS	Le Busseau	44	6.74	0.07	0.76	0.006
	CHE	La Chapelle‐Saint‐Etienne	41	6.35	0.02	0.75	0.006
	FAY	Faye l'Abesse	76	6.57	0.04	0.75	0.032
	FEN	Fenioux	87	6.51	0.04	0.75	−0.006
	LES	Lessac	45	6.59	0	0.75	0.022
	LOU	Saint‐Loup‐sur‐Thouet	19	6.54	0.02	0.73	0.030
	PIN	Le Pin	49	6.57	0.01	0.74	0.002
	XAI	Xaintray	77	6.77	0.04	0.76	0.002
	ARG	Argelouse	29	6.48	0.01	0.75	−0.001
	SAR	Sarran	77	6.78	0.04	0.76	0.008
	VIG	Vignols	41	6.45	0.03	0.75	−0.0001
	BED	Bedous	25	6.18	0.05	0.74	0.003
	LAC	Lacanau	15	6.96	0.03	0.73	0.039
	LAN	Langeac	25	6.31	0.04	0.74	−0.021
	AYD	Aydat	21	6.33	0	0.72	0.060
	KER	Kernascleden	25	6.08	0.03	0.74	−0.013
	LYS	Lys‐Haut‐Layon	19	6.72	0	0.75	0.020
	BER	Bernières d'Ailly	24	6.70	0.01	0.75	−0.030
	ARL	Arles	25	6.77	0.08	0.75	−0.022
	MON	Montreuil‐sur‐mer	15	5.53	0	0.67	−0.037
Spanish Basque Country	LEZ	Lezate	28	6.35	0.05	0.73	0.020
England	BRY	Bryanston	19	4.61	0	0.59	−0.004
	BUC	Buckfastleigh	17	4.90	0.07	0.60	0.120
North Africa	GHA	El Feidja National Park	22	6.69	0.97	0.68	0.002

Note

Sample size (*N*), corrected allelic richness (*A*_r_), corrected private allele richness (*pA*), expected heterozygosity (*H*_e_), and inbreeding coefficient (*F* _is_).

For each bat, a tissue sample was collected from the wing membrane (patagium) using a 3‐mm diameter biopsy punch. Samples were either preserved in 95° ethanol and quickly stored at 4°C until DNA extraction or preserved in silica‐gel (Puechmaille et al., 2011). Biopsy punches were cleaned with bleach, water, and then ethanol between each sample collection.

### 2.3 DNA extraction and microsatellite genotyping

DNA was extracted from each wing sample using the EZ‐10 Spin Column Genomic DNA Minipreps Kit for Animal (BioBasic) following the manufacturer's instruction with a final elution of twice 50 µl in elution buffer. We amplified 17 microsatellite loci using primers modified from those previously designed for *R. ferrumequinum* (Dawson, Rossiter, Jones, & Faulkes, 2004; Rossiter, Burland, Jones, & Barratt, 1999) and the lesser horseshoe bat (*Rhinolophus hipposideros*; Puechmaille, Mathy, & Petit, 2005; Table S1). Amplifications were organized into three multiplex PCRs that were conducted in 10 µl reaction volumes containing 5 µl of the QIAGEN Multiplex PCR Master Mix (1×) (including *Taq*, dNTPs, and 3 mM of Mg_2+_ as final concentration), 2 µl of extracted DNA, and 0.2 µl of each primer (0.2 µM as final concentration). We used the following PCR conditions: 95°C for 15 min, 35 cycles of 94°C for 30 s, 57°C for 90 s and 72°C for 60 s, and a final elongation step at 60°C for 30 min. Twenty microliters of H_2_O was added to 10 µl of each product in a new plate, and 2 µl of this final solution was mixed with 15 µl of formamide and 0.08 µl of GeneScan 500 LIZ. The samples were genotyped using an ABI3130 automated sequencer and the GeneMapper v.5.0 software. Two independent readings were performed by different people to minimize genotyping errors.

Null alleles, large allele dropout, stuttering, and scoring inconsistencies were tested for each colony using MICROCHECKER (Van Oosterhout, Hutchinson, Wills, & Shipley, 2004). Null allele frequencies were estimated for each locus and population using FreeNA, with 1,500 replicates for the computation of the bootstrap 95% confidence intervals (Chapuis & Estoup, 2007). We performed the exact tests implemented in GENEPOP v4.6 (Rousset, 2008) to detect linkage disequilibrium for each pair of loci in each colony and to investigate departure from Hardy–Weinberg equilibrium (HWE) for the whole dataset and for each colony and for each locus under the hypothesis of heterozygote deficit. We used the false discovery rate (FDR) to account for multiple testing (Benjamini & Hochberg, 1995). The adjusted *p*‐value thresholds after FDR correction (*p*_critical_) were calculated following Castro and Singer (2006).

### 2.4 Genetic diversity and relatedness within colonies

We assessed genetic diversity within colony by estimating the allelic richness and private allele richness corrected for minimal sample size (*A*_r_ and pA, *N* = 13), the expected (*H*_e_) heterozygosity, using FSTAT v.2.9.3.2 (Goudet, 1995), HP‐RARE (Kalinowski, 2005), and GENETIX v.4.05.2 (Belkhir, Borsa, Chikhi, Raufaste, & Bonhomme, 2004), respectively. Differences in *A*_r_ and *H*_e_ between colonies were evaluated using the comparison among groups of samples implemented in FSTAT with 1,000 permutations. The adjusted *p*‐value thresholds after FDR correction (*p*_critical_) were calculated following Castro and Singer (2006).

We estimated the fixation index *F*_IS_ (Weir & Cockerham, 1984) using GENEPOP v4.6. We next performed relatedness analyses to verify whether population genetic structure could not be due to the comparison of different family units rather than populations (Schweizer, Excoffier, & Heckel, 2007). We estimated the maximum likelihood pairwise coefficient of relatedness *r* between pairs of individuals on an absolute scale (0, unrelated to; 1, identical individuals), using the ML‐RELATE software (Kalinowski, Wagner, & Taper, 2006). Indeed maximum likelihood estimate is less biased than commonly used estimators (Milligan, 2003). The coefficient *r* corresponds to the probability for each locus that individuals share zero, one, or two alleles that are identical by descent. We used the genetic clusters identified from further clustering analyses (see below) as population references for estimating *r* within each colony.

### 2.5 Population structure and genetic differentiation between colonies

The genetic differentiation between colonies was quantified using estimates of global and pairwise *F*_ST_ (Weir & Cockerham, 1984). Significance was assessed using exact G‐test of differentiation implemented in GENEPOP v4.6 (Rousset, 2008). Pairwise *F*_ST_ and exact G‐tests were computed for each pair of colonies and each pair of genetic clusters identified from further clustering analyses. Analyses were performed with and without juveniles. We accounted for multiple testing using false discovery rate (FDR). In order to control for potential effects of null alleles on genetic differentiation, we also estimated pairwise *F*_ST_ corrected for null alleles using the “Excluding Null Alleles” (ENA) correction implemented in FreeNA (Chapuis & Estoup, 2007).

Genetic structure was also investigated using several complementary approaches to give a robust cross‐validation of our results and prove that the observed genetic signature is robust despite the potential violation of the underlying hypothesis. First, we used the clustering approach implemented in the STRUCTURE program v2.3.4 (Pritchard, Stephens, & Donnelly, 2000) to determine the presence of genetic discontinuities without any a priori knowledge. We determined the most likely number of genetic clusters using the log‐likelihood of *K* and ∆*K* statistic (Evanno, Regnaut, & Goudet, 2005) implemented in the website STRUCTURE HARVESTER (Earl & vonHoldt, 2012). We used the admixture model with uncorrelated frequencies and an alpha‐value of 1/*K*, as recommended by Wang (2017) in the case of unbalanced sampling. The same results were obtained with the default alpha‐value and when applying or not the LOCPRIOR model to the population model (Hubisz, Falush, Stephens, & Pritchard, 2009) and were therefore not presented here. Puechmaille (2016) demonstrated that the ∆*K* statistic was biased in the case of uneven sampling (as in our current study). However, in the present study, given the fact that three recovered clusters (see Section 3) were geographically coherent, were composed of colonies with nonsignificant or very low *F*_ST_ values (see Section 3), and were consistent with previous genetic findings, we did not perform further subsampling or used alternative estimators. We performed 20 independent runs with a burn‐in period of 1,000,000 iterations and 50,000 MCMC repetitions after burn‐in, testing *K* = 1 to *K* = 28 (*N* colonies + 1). We used the R package *pophelper* (Francis, 2017) to compute the plots. In addition, we also performed a principal component analysis (PCA) implemented in the R packages *ade4* (Dray & Dufour, 2007) and *factoextra* (Kassambara & Mundt, 2017). Contrary to STRUCTURE algorithm, this approach does not rely on any specific population genetic assumption including Hardy–Weinberg equilibrium and linkage equilibrium (Pritchard et al., 2000).

We next used MAPI program (Mapping Averaged Pairwise Information, Piry et al., 2016), implemented in the R package *mapi*, to detect spatial genetic discontinuity. This approach has low sensitivity to potential confounding effects resulting from isolation by distance (IBD) and does not require predefined population genetic model. It is based on a spatial network in which pairwise genetic distance between georeferenced samples is attributed to ellipses. A grid of hexagonal cells covers the study area and each cell receives the weighted arithmetic mean of the pairwise genetic distance associated to the ellipses intersecting the cell (Piry et al., 2016). We used the Rousset's coefficient *â* (Rousset, 2000) computed with SPAGeDi 1.4 (Hardy & Vekemans, 2002) as an index of pairwise genetic differentiation between individuals and default parameter value for the eccentricity of the ellipses (0.975). We used the permutation procedure (1,000 permutations) to identify areas exhibiting significantly higher or lower levels of genetic differentiation than expected by chance.

Lastly, isolation by distance (IBD) was analyzed with the regression of the genetic distances between colonies (*F*_ST_/1−*F*_ST_; Rousset, 1997) and the logarithm of the Euclidean geographic distances, implemented in GENEPOP v4.6. Confidence intervals and significance of regression slope and intercept were assessed by bootstrapping over loci. It was then tested using Mantel tests with 10,000 permutations. IBD analyses were performed on the complete dataset, within France and between pairs of colonies sampled in the different countries. The comparison of the results gathered from these IBD analyses should enable the identification of geographic barriers (Pyrenees mountain range, Channel Sea, and Mediterranean Sea) that might reduce *R. ferrumequinum* gene flow.

### 2.6 Inference of demographic parameters

We first inferred the demographic history of *R. ferrumequinum* colonies. We used the software MIGRAINE v.0.5.1 (Leblois et al., 2014) and the model *OnePopVarSize*. This model assumes a unique variation starting *T* generations ago in population size of an isolated panmictic population that was followed by continuous exponential change in population size until the moment of sampling. This model estimates three scaled parameters: the current scaled population size (*θ* = 4*N*_e_ *µ*) and the ancestral scaled population size (*θ*_anc_ = 4*N*_anc_ *µ*). *N*_e_ and *N*_anc_ are the current and ancestral diploid population sizes, *µ* is the mutation rate per generation of the loci, and *D* is the time scaled by current population size (*D* = *T*/4*N*) at which an already existing population experienced a demographic change. The mutation model used was a generalized stepwise mutation model (GSM) which is characterized by a geometric distribution of mutation steps with the parameter pGSM. We used the ratio *Nratio* (*N*/*N*_anc_) to characterize the strength of demographic events. In the case of a contraction, the *Nratio* is <1, and in the case of an expansion, it is >1. The change in population size was considered significant when the 95% confidence intervals (95% CIs) of the *Nratio* did not include the value “1.” Runs were performed independently for each colony and for each genetic cluster that was previously identified. We used 1,000 to 20,000 trees per point, 600 to 800 points, and eight iterations by run. When no signal of demographic change was found, we used the *OnePop* model implemented in MIGRAINE to estimate the scaled effective population size *θ* = 4*N*_e_ *µ* of stable and panmictic population.

We then inferred contemporary levels and directions of migration between the main genetic clusters using the program BAYESASS v3.0.4 (Wilson & Rannala, 2003). We performed five independent runs of 10,000,000 iterations sampled every 2000 iterations, with a burn‐in of 1,000,000. For each run, we calculated the Bayesian deviance using the R script provided by Meirmans (2014). We used this deviance as a criterion to find the run that provided the best fit and to identify runs with convergence problems (Faubet, Waples, & Gaggiotti, 2007; Meirmans, 2014). We ran preliminary runs to adjust the maximum parameter change per iteration (Delta values). It is important to optimize the acceptance rates for proposed changes to parameters (20% to 60% is ideal; Wilson & Rannala, 2003). The adjustments are important because if the acceptance rates are too low or too high, the chain does not mix well and fails to adequately explore the state space. In the final run, we used delta values of 0.45, 0.40, and 0.51 for allele frequency, migration, and inbreeding, respectively.

## 3 RESULTS

Three of the 17 microsatellites genotyped were excluded from further genetic analyses: one of them was monomorphic and the other two had poor quality profiles. Results gathered using MICROCHECKER showed no large allele dropout or scoring inconsistencies due to stuttering. Null alleles were suspected at loci Rferr06 in three colonies from France (“AIR,” freq = 0.074; “FEN,” freq = 0.046; “XAI,” freq = 0.041), and at loci RHD103 (France, “ARL,” freq = 0.078), Rferr27 (France, “BED,” freq = 0.152), and Rferr01 (England, “BUC,” freq = 0.149). Nevertheless, we did not detect any deviation from HWE in any colony. Fifteen out of the 2,418 pairs of loci (0.62%) exhibited significant linkage disequilibrium, but the loci involved were not consistent among colonies. Therefore, we did not exclude any other loci.

### 3.1 Genetic diversity and relatedness

**Figure 2:**
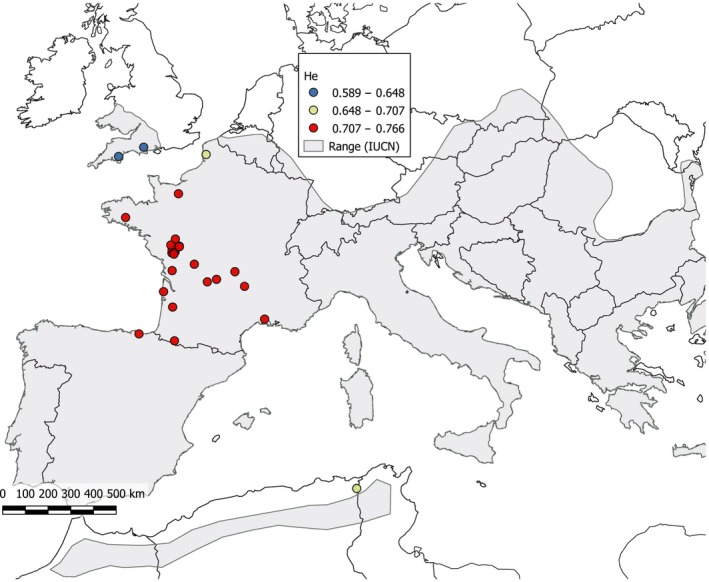
Expected heterozygosity (*H*_e_) estimated for each colony. Blue to red colors indicate low to high levels of *H*_e_. The geographic range of *R. ferrumequinum* is shaded in gray

A summary of the genetic diversity indices (*F* _is_, *A*_r_, *H*_e_) based on the 14 validated microsatellites is presented in Table 1. Global Hardy–Weinberg test (H1 = heterozygote deficit) was significant (*p* = .017, *F* _is_ = 0.009). Local *F* _is_ estimates (i.e., for each colony) ranged from −0.037 (“MON,” northern France) to 0.120 (“BUC,” England). All tests of departure from local HWE were nonsignificant after FDR correction (adjusted *p* > *p*_critical_). The English colonies showed significant lower estimates of allelic richness (adjusted *p* = .004 < *p*_critical_ = .012) and expected heterozygosity (adjusted *p* = .030 < *p*_critical_ = .037) than the French ones (Table 1, Figure 2). The northern French colony “MON” showed lower allelic richness estimate than the other French colonies (adjusted *p* = .030 < *p*_critical_ = .037) but similar *H*_e_ estimate (adjusted *p* = .059 > *p*_critical_ = .037). The Tunisian colony “GHA” showed similar levels of allelic richness and expected heterozygosity than the French colonies (adjusted *p* > *p*_critical_). Private allelic richness was low in all colonies except the Tunisian one (“GHA”; Table 1).

**Figure 3:**
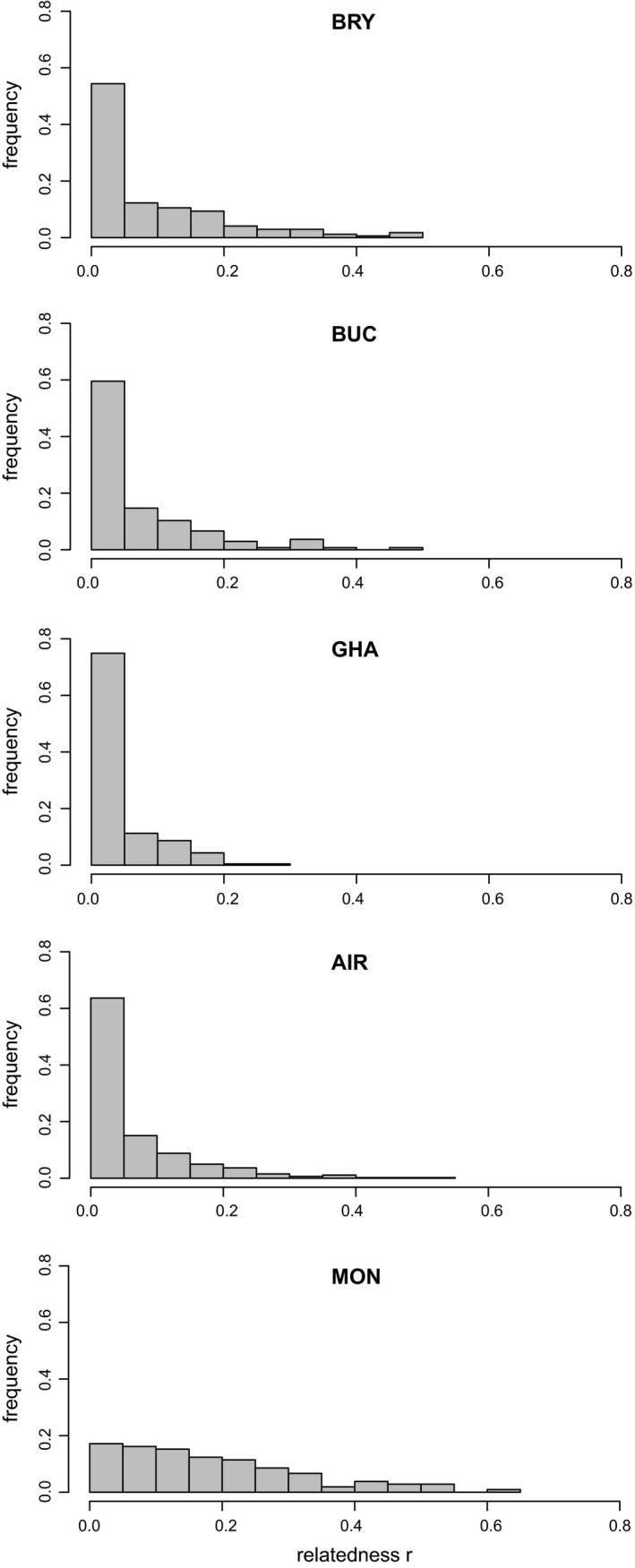
Distribution of the pairwise relatedness coefficient *r* estimated with the ML‐relate software (Kalinowski et al., 2006) within colonies from France (AIR, MON), England (BRY, BUC), and Tunisia (GHA)

**Figure 4:**
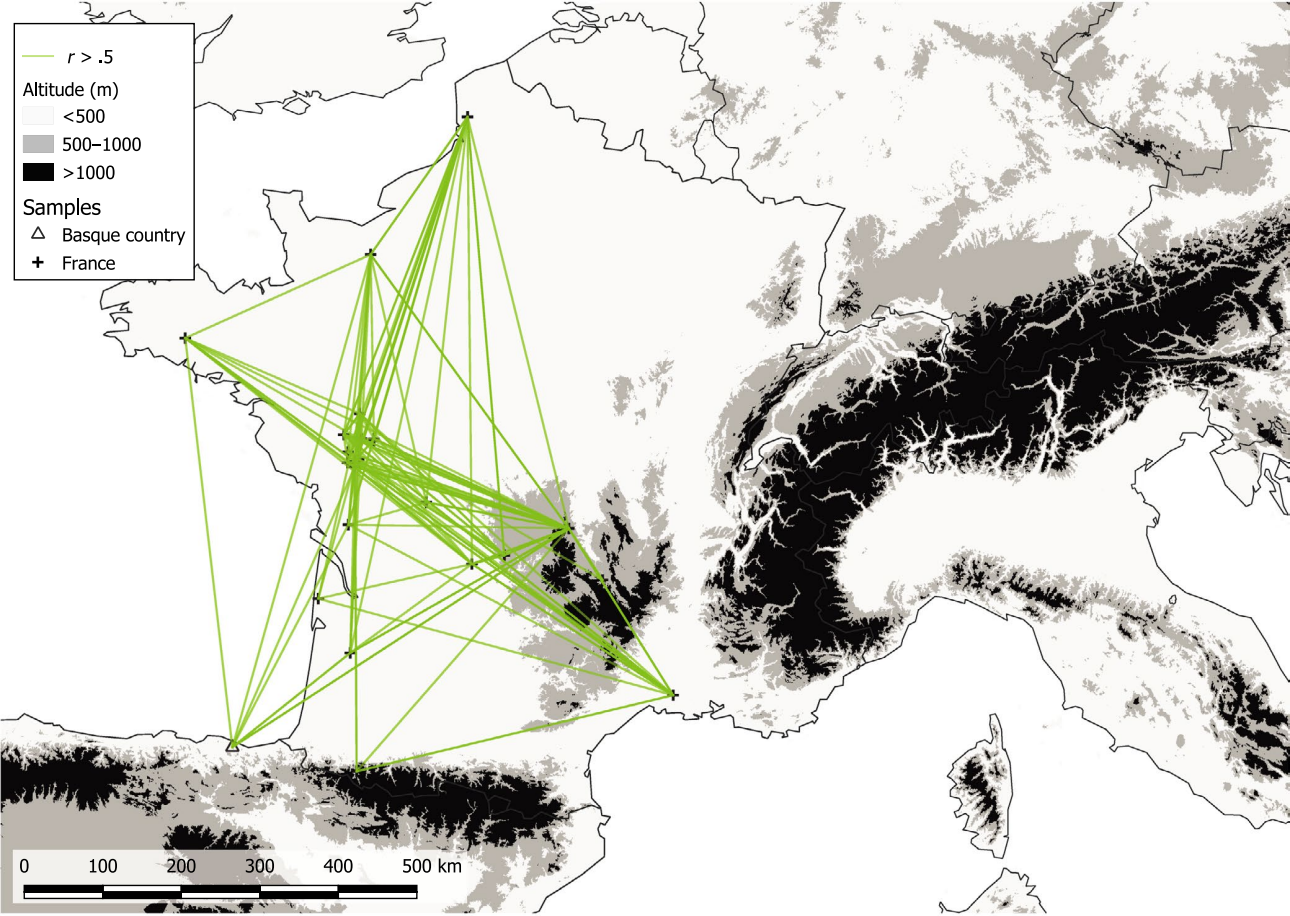
Geographic distribution of the higher values of pairwise relatedness coefficient (*r* > .5) between *R. ferrumequinum* colonies from the continental genetic cluster (France and the Spanish Basque Country)

The distributions of the pairwise coefficient of relatedness *r* within each colony showed a common pattern in all colonies except in the northern French colony “MON” (Figure 3). For all colonies, the distribution of the *r* coefficient was L‐shaped with a peak of unrelated individuals (*r* = 0) and a decreasing proportion of related individuals. In the northern French colony “MON,” we observed a more uniform proportion of unrelated and relatively closely related individuals (*r* ranging between .0 and .3). When considering pairwise relatedness between individuals from different colonies, we still observed high levels of relatedness (*r* > .5; Figure 4). The high levels of relatedness involved female–female pairs and female–male pairs but never male–male pairs. It concerned 537 females, 33 males, and one undetermined individual. The majority of the females were adults (474 adults, 59 juveniles, and four undetermined), but this was not the case when considering males (12 adults, 19 juveniles, and two undetermined).

### 3.2 Population structure and genetic differentiation between colonies

*F*_ST_ estimates calculated with and without excluding null alleles (ENA) were very similar, and analyses without juveniles did not substantially change *F*_ST_ estimates. Therefore, we only reported the uncorrected estimates for the dataset including juveniles. The pairwise *F*_ST_ estimates between colonies and associated *G*‐tests are presented in Table S2. Low but significant genetic differentiation was observed between colonies within western France (*F*_ST_ < 3%, 65.24% of the G‐tests with *p* < .05; Table S2). The northern French colony “MON” exhibited higher estimates of pairwise *F*_ST_ than the other colonies (3.49% < *F*_ST_ < 6.46%, *G*‐tests *p* < 10^−3^). Among the French colonies, “MON” was also the colony that exhibited the highest levels of genetic differentiation with the English (*F*_ST_ > 16%, *G*‐tests *p* < .001), Spanish Basque (*F*_ST_ = 7.17%, *G*‐tests *p* < .001), and Tunisian colonies (*F*_ST_ = 17.05%, *G*‐tests *p* < .001). The Spanish Basque colony “LEZ” was more genetically differentiated from colonies in England and Tunisia (England: 9.88% < *F*_ST_ < 11.9%, *G*‐tests *p* < .001 and Tunisia: *F*_ST_ = 14.87%, *G*‐tests *p* < .001) than from colonies in France (*F*_ST_ < 3%).

**Figure 5:**
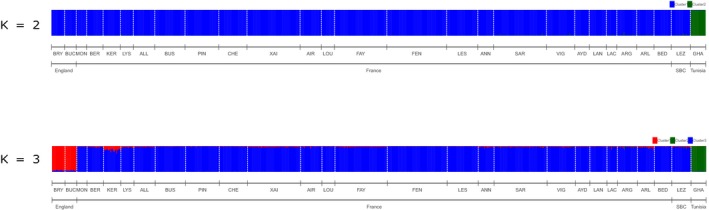
STRUCTURE plots for *K* = 2 and *K* = 3 clusters. Each bar represents an individual colored according to its membership probability to a given cluster. Individuals are sorted by localities. The white dashed lines separate the 27 colonies where the individuals were sampled: Bryanston (BRY), Buckfastleigh (BUC), Montreuil‐sur‐mer (MON), Kernascleden (KER), Lys‐Haut‐Layon (LYS), Allonne (ALL), Le Busseau (BUS), Le Pin (PIN), La Chapelle‐Saint‐Etienne (CHE), Xaintray (XAI), Airvault (AIR), Saint‐Loup‐sur‐Thouet (LOU), Faye l'Abesse (FAY), Fenioux (FEN), Lessac (LES), Annepont (ANN), Sarran (SAR), Vignols (VIG), Aydat (AYD), Langeac (LAN), Lacanau (LAC), Argelouse (ARG), Arles (ARL), Bedous (BED), Lezate (LEZ), and El Feidja National Park (GHA). Abbreviation: SBC, Spanish Basque Country

Using STRUCTURE, we found that the ∆*K* statistics (Evanno et al., 2005) were highest for *K* = 2, but the likelihood of the number of genetic clusters Ln (L(*K*)) showed similar values for *K* = 2 and *K* = 3 (Figure S1). For *K* = 2, the first genetic cluster included all colonies from England, France, and Spanish Basque Country and the second genetic cluster included the Tunisian colony. For *K* = 3, the first main genetic cluster included all colonies from western France and Spanish Basque Country. The second main genetic cluster included the two colonies from England and the third genetic cluster included the Tunisian colony (Figure 5). Increasing *K* did not change this clustering pattern. The PCA showed similar results, with three main genetic clusters (England, western France, Spanish Basque Country, and Tunisia, Figure S2).

**Figure 6:**
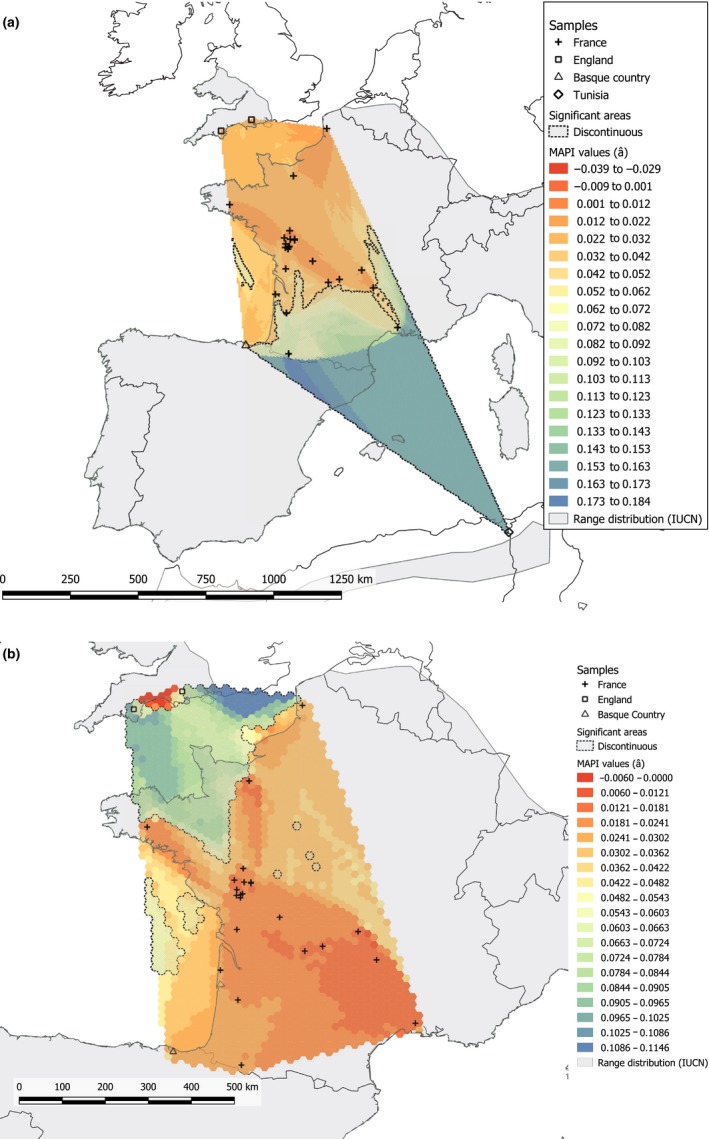
Geographic distribution of the pairwise Rousset's genetic distance â (Rousset, 2000) resulting from MAPI (Piry et al., 2016). (a) All colonies are included. (b) The Tunisian colony is not included in the analysis. Dots correspond to the colonies sampled in this study. Levels of genetic dissimilarity are indicated using a color scale ranging from red (lower genetic dissimilarity) to blue (higher genetic dissimilarity). Significant areas are represented by dashed lines

While including all colonies, the program MAPI revealed that the Mediterranean Sea corresponded to an area of significantly higher genetic dissimilarity (Figure 6a). A second analysis excluding the Tunisian colony highlighted a significantly higher genetic dissimilarity between the English colonies on the one hand and the French and Basque colonies on the other. It also showed the existence of genetic homogeneity in the continent (Figure 6b).

**Figure 7:**
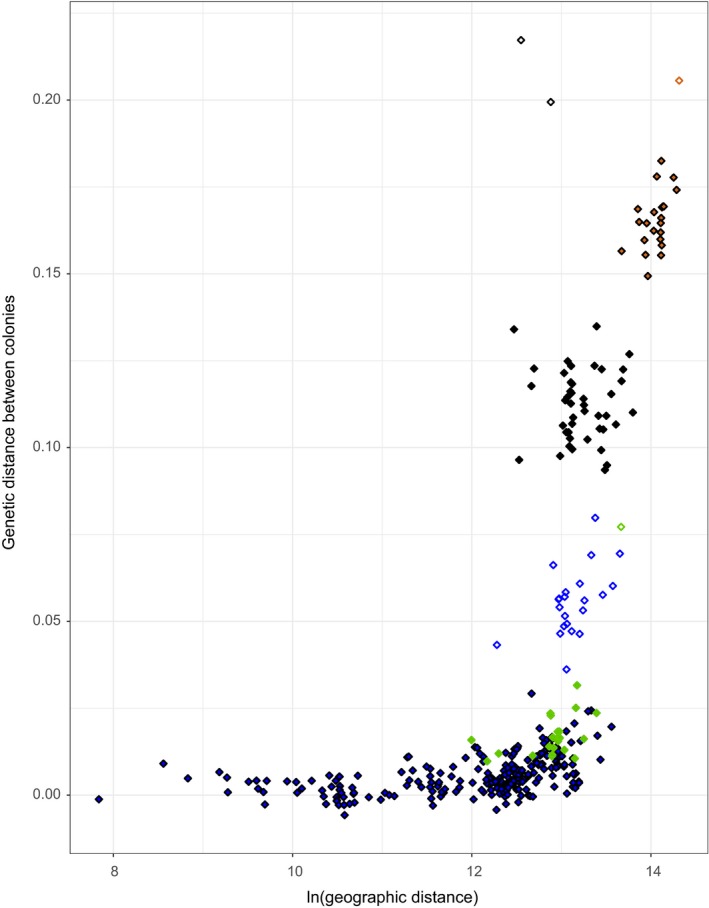
Plots of the within/between isolation by distance analyses. The genetic distance between colonies was estimated as (*F*_ST_/1 − *F*_ST_). The color of the dots indicates the geographic origin of the pairs of colonies: within France (blue), between France and Spanish Basque Country (green), between France and Tunisia (brown) and between France and England (black). The pairs of colonies that include the French colony “MON” are not color filled

We found a significant positive relationship between genetic differentiation and geographic distance within France (slope of the regression = 0.0063 [0.0042–0.0090]; *p*_Mantel_ < .001), between France and Spanish Basque Country (slope of the regression = 0.0212 [0.0120, 0.0412]; *p*_Mantel_ < .001), and between France and Tunisia (slope of the regression = 0.0457 [0.0009, 0.1390]; *p*_Mantel_ = 10^−3^). The northern French colony “MON” was more genetically differentiated from the other French colonies (Figure 7). When excluding this particular colony “MON” from the regression analyses, we still found a positive relationship between genetic differentiation and geographic distance among French colonies but the slope was weaker (slope of the regression = 0.0025 [0.0015–0.0036]; *p*_Mantel_ < .001). Moreover, considering only differentiation between pairs for French–English, French–Basque, and French–Tunisian colonies without including the northern French colony “MON,” the isolation by distance relationship was not significant (Table 2). All results are presented in Table 2 and Figure 7.

**Table 2:** Isolation by distance characteristics using the genetic differentiation parameter (*F*_ST_/1−*F*_ST_) between colonies against the logarithm of the Euclidian geographical distance

	Geographic zone	Intercept [CI]	Slope [CI]	*p* Mantel test
Including “MON”	All datasets	−0.3395 [−0.5402, 0.2411]	0.0303 [0.0217, 0.0475]	0***
	Within France	−0.0667 [−0.0963, −0.0428]	0.0063 [0.0042, 0.0090]	0***
	Between France and England	0.4177 [0.0645, 0.9419]	−0.0229 [−0.0603, 0.0025]	.912
	Between France and Tunisia	−0.4755 [−1.7182, 0.1382]	0.0457 [0.0009, 0.1390]	.001***
	Between France and SBC	−0.2547 [−0.5020, −0.1373]	0.0212 [0.0120, 0.0412]	.0001***
Excluding “MON”	All datasets	−0.3328 [−0.5279, −0.2406]	0.0296 [0.0217, 0.0463]	0***
	Within France	−0.0242 [−0.0357, −0.0142]	0.0025 [0.0015, 0.0036]	0***
	Between France and England	0.1304 [−0.1185, 0.4372]	−0.0014 [−0.0229, 0.0166]	.563
	Between France and Tunisia	−0.2265 [−0.7600, 0.2584]	0.0279 [−0.0050, 0.0687]	.013*
	Between France and SBC	−0.0774 [−0.1841, 0.0483]	0.0073 [−0.0021, 0.0158]	.010*

Note

95% confidence intervals (CI) for the slope and the intercept of the IBD were obtained by ABC bootstrapping. Significant *p*‐values of Mantel tests are represented in bold with *** for *p* < .001, ** for *p* < .01, and * for *p* < .05. “MON” is the northern French colony of the study area.

Abbreviation: SBC, Spanish Basque Country.

### 3.3 Inference of demographic parameters

Using MIGRAINE, we detected a significant signature of expansion for the Tunisian colony (“GHA”; *N*_ratio_ = 2.989 [1.63–8063]), with an estimated origin of this demographic change *D* = 0.811 [2.17 e−06–2.201]. None of the other colonies or genetic clusters showed significant signature of demographic change (Table S3). The marginally significant signature of contraction observed for the western French colony “CHE” in the Poitou‐Charentes region was also considered to be nonsignificant because the higher value of the confidence interval was very close to 1 (0.99) and because we performed a high number of tests.

**Figure 8:**
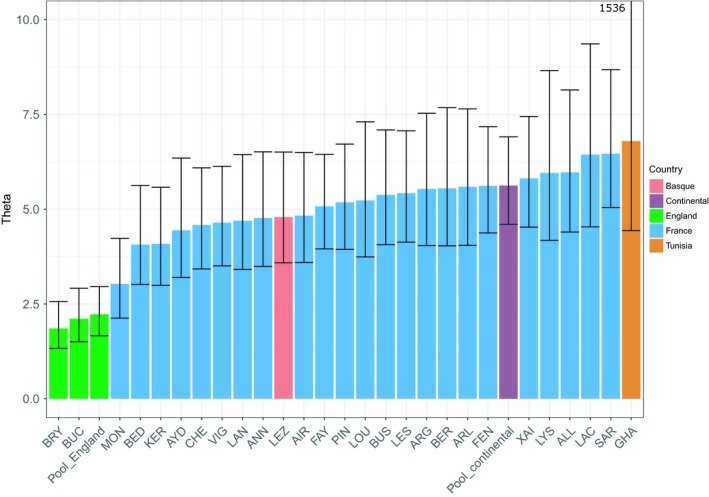
Estimation of *θ* (4*N*_e_ *µ*) for each colony and genetic cluster based on the OnePop model for stable colonies and OnePopVarSize for the Tunisian colony “HA” which experienced expansion. As the confidence interval of Tunisian *θ* is very large (4.435–1536), the superior born was not represented to avoid the flattening of the graph. Pool_England and Pool_continental respectively correspond to the English and French‐Spanish Basque colonies. Colonies are classified by *θ* estimates

We estimated the scaled current population size *θ* (4*N*_e_ *µ*) for all stable colonies, and *θ* estimates ranged between 1.854 and 6.465 (Table S4, Figure 8). The lowest estimates were found for the English colonies (“BRY” *θ* = 1.854; “BUC” *θ* = 2.107) and for the northern French colony “MON” (*θ* = 3.030). Other colonies and pool of colonies exhibited similar levels of *θ* estimates (from 4.066 to 6.465). Kruskal–Wallis tests and post‐hoc pairwise comparisons of *θ* estimates using Wilcoxon test (with FDR correction for multiple testing) revealed a significant difference of *θ* estimates between the western French–Spanish Basque and the English genetic clusters (*p* = .002) and nonsignificant differences between the other clusters (*p* > .115). We observed a significant negative relationship between the estimated *θ* of each colony and the distance of the colony to the centroid of our sampling (*p* < .05; Figure S3).

The five runs implemented in BAYESASS provided similar results and converged well, with values ranging from *D*_run2_ = 67,128.6 to *D*_run4_ = 67,134.7. None of the migration rates estimated between the three main genetic clusters was significant (Table 3). Within the western French–Spanish Basque cluster, we found significant unidirectional migration rates from the French to Spanish Basque colonies (*m* = 0.2921 ± 0.0192). As our results emphasized particular signatures for the northern French colony “MON” all along the study, we also estimated migration rates considering “MON” apart from the other French colonies. Our results revealed significant unidirectional migration rates from all western French colonies to the northern French colony “MON” (*m* = 0.2700 ± 0.0276).

**Table 3:** Means of the posterior distributions of contemporary migration rate (m) with standard deviation in parentheses

Migration into	Migration from				
	Genetic cluster 1			Genetic cluster 2	Genetic cluster 3
	MON	France	Basque	England	Tunisia
MON	**0.6825 (0.0152)**	**0.2700 (0.0276)**	0.0161 (0.0153)	0.0158 (0.0148)	0.0156 (0.0153)
France	0.0004 (0.0004)	**0.9975 (0.0013)**	0.0004 (0.0004)	0.0012 (0.0011)	0.0004 (0.0004)
Basque	0.0101 (0.0095)	**0.2921 (0.0192)**	**0.6768 (0.0101)**	0.0109 (0.0108)	0.0102 (0.0100)
England	0.0082 (0.0081)	0.0128 (0.0116)	0.0083 (0.0082)	**0.9626 (0.0169)**	0.0080 (0.0078)
Tunisia	0.0129 (0.0125)	0.0126 (0.0121)	0.0129 (0.0122)	0.0124 (0.0119)	**0.9493 (0.0229)**

Note

Migration and nonmigration rates that are significantly > 0 are represented in bold.

## 4 DISCUSSION

### 4.1 A large and stable population of *R. ferrumequinum* ranging from Spanish Basque Country to northern France

Our study revealed high and homogeneous levels of genetic diversity within the western French and Spanish Basque colonies examined. These levels were similar to those previously detected in colonies from the eastern part of *R. ferrumequinum* distribution (Rossiter et al., 2007, 2000), or in other bat species such as *Rhinolophus euryale* and *Myotis myotis* (Budinski et al., 2019; Castella, Ruedi, & Excoffier, 2001). We did not find evidence of departure from Hardy–Weinberg equilibrium within colonies or when considering the western French and Spanish Basque colonies altogether. We also found very low levels of genetic differentiation between these colonies of *R. ferrumequinum*, as indicated by *F*_ST_ estimates. These colonies were identified as a unique genetic cluster according to the results provided by STRUCTURE and MAPI analyses and the slight isolation by distance pattern detected which suggests that some admixture occurs at long‐distance, thus interconnecting the sampled localities. Altogether, these results indicated high gene flow between colonies at this scale and suggested that the western French and Spanish Basque colonies form a single and large population. In that respect, our results supported the previous studies of *R. ferrumequinum* phylogeography which had revealed a unique genetic cluster in Western Europe (Switzerland apart, Flanders et al., 2009; Rossiter et al., 2007). In addition to these findings, we could infer the scaled effective size *θ* (4*N*_e_ *µ*) of this population. We found homogeneous *θ* among the western French and Spanish Basque colonies and for the pool of these colonies. This result was congruent with our finding of one large population with each colony representing a replicate of the whole population. We could not transform this estimate *θ* into an effective number of individuals (*N*_e_) because it requires fixing the mutation rate of microsatellite markers in this species. This is a crucial issue because estimates of mutation rates can greatly vary between and within species and any inaccuracy in the mutation rate estimate is propagated in the estimation of *N*_e_ (Waples, 2010).

We did not reveal any signature of demographic decline, neither for the colonies of the French Poitou‐Charentes region nor for the delineated western French and Spanish Basque population of *R. ferrumequinum*. Several biases could have lead to the nondetection of a demographic event. First, Leblois et al. (2014) showed that the capacity to detect demographic events from genetic data depends on the number of genetic markers used, the strength of the event, and the time when it happened. Therefore, because the decline was recent and we used 14 microsatellites, our study may have suffered from a lack of power that could explain the absence of signature of demographic decline in our data. Second, the maximum lifespan of these bats is 30 years (Caubère, Gaucher, & Julien, 1984), and simulations of microsatellite data analyzed under similar conditions using MsVar (Beaumont, 1999; Storz & Beaumont, 2002) on the eastern red bat *Lasiurus borealis* have shown a significant delay in the response of coalescent‐based *N_e_* estimates to recent population declines (Munster, 2015). However, our results are congruent with the very recent analysis of the historical Poitou‐Charente roost counts, which revealed a stable population in this region over the past 15 years (M. Leuchtmann, personal communication).

### 4.2 Dispersal and reproduction of *R. ferrumequinum*

Our results revealed that the Mediterranean Sea and the English Channel may act as barriers to *R. ferrumequinum* gene flow. Seas have often been shown to limit bat gene flow, irrespectively of bat flight capacity (García‐Mudarra et al., 2009). The high genetic differentiation observed between the English colonies in this study was expected because of the potential founder effects associated with *R. ferrumequinum* colonization since the Late Glacial Maximum (LGM; Flanders et al., 2009; Rossiter et al., 2007). Our demographic inferences also revealed the absence of current gene flow between the two English and western French–Spanish Basque clusters. The high genetic differentiation observed between Tunisian and western French–Spanish Basque colonies may also rely on historical colonization history. *R. ferrumequinum* seems to have been present in North Africa before the Late Glacial Maximum (Flanders et al., 2009; Rossiter et al., 2007), and there is no evidence that North Africa was recolonized from Europe post‐LGM. These historical patterns of genetic differentiation might have been reinforced by the current absence of gene flow between Europe and North Africa. However, our sampling is too sparse to assess gene flow over this large landscape feature, and we cannot exclude the possibility that the Strait of Gibraltar, which leaves a gap of just 14 km, connects populations of *R. ferrumequinum* across the Mediterranean Sea. The degree of permeability of the Mediterranean Sea for this bat species therefore deserves a dedicated study, aiming at assessing the potential pathways of *R. ferrumequinum* movements and gene flow between Europe and North Africa.

Similarly, as we only had a few samples from both sides of the Pyrenees, we could not assess the potential effect of this mountain as a barrier to gene flow. The absence of (or weak) gene flow disruption associated with western Pyrenees in this study may therefore suggest that this mountain may not impede dispersal or that *R. ferrumequinum* uses the shoreline as corridor along both the Atlantic and Mediterranean edges of the Pyrenees, where altitude is lower than 500 m, as has been seen for migratory birds (Galarza & Tellería, 2003). The situation is different from another *Rhinolophus* species, *R. hipposideros*, where the Pyrenees most likely act as a strong barrier (Dool et al., 2013). Future genetic and ecological studies including dense sampling from both sides and all along the Pyrenees are now required to assess whether *R. ferrumequinum* movements and genetic mixing are restricted by mountains.

In the absence of important landscape barriers such as seas or, potentially, mountains, we showed that *R. ferrumequinum* is able to move over hundreds of kilometers, as exemplified by the low levels of genetic differentiation observed at large geographical scales, the inference of significant migration rates between the western French–Spanish Basque maternity colonies, and the high levels of relatedness observed between individuals sampled in distant colonies.

These results also revealed the high genetic mixing that occurs at large scale between *R. ferrumequinum* western French and Spanish Basque colonies. Several demographic processes may underlie this genetic mixing. First, mating dispersal at large distance would lead to extracolony copulations and to the relaxation of colonies' genetic borders (Veith, Beer, Kiefer, Johannesen, & Seitz, 2004). Second, because we also found high levels of relatedness between juveniles (under two years old) sampled at considerable distances (up to 861 km), natal dispersal (one‐way movement of juveniles during their first year, from their colony of birth to another) and/or movements of adults from one maternity colony to another between two consecutive reproduction events (years) are also potential mechanisms shaping genetic mixing. These alternatives are still difficult to evaluate due to a lack of knowledge with regard to *R. ferrumequinum* mating behavior (where and when) and dispersal, in particular the one of males. Ringing data from *R. ferrumequinum* from across Europe suggest the species is mostly sedentary but with occasional movements over 100 km. Indeed, although rare, there are documented movements of 180 km in Spain, 320 in Hungary (reviewed in Hutterer, Ivanova, Meyers‐Cords, & Rodrigues, 2005), and 500 km in France (Saint Girons, 1973), clearly demonstrating the species is occasionally able to move over large distances. In the future, long‐term capture‐mark‐recapture surveys of adults and juveniles could provide invaluable information to assess the relative importance of mating, natal, and breeding dispersal in the genetic mixing of colonies within management units. Given the scale at which the species is suspected to move given the available ringing data and our current genetic results (weak population structure), it would be important to monitor sites (for recaptures) not only close to the ringing sites but also several hundreds of kilometers away. This will make these studies, which would ideally be long‐term studies, logistically challenging.

### 4.3 Differences in the functioning of central–peripheral and island–continental colonies

Our results revealed contrasting patterns of genetic structure within and between populations when comparing *R. ferrumequinum* western French–Spanish Basque population with colonies from Tunisia, England, and northern France (“MON”). In these latter colonies, we detected lower levels of genetic diversity (*H*_e_ up to 20% lower) and smaller estimates of *ϴ* (4*N*_e_ *µ*; up to half the size) than in the western French–Spanish Basque population. They were also more genetically differentiated than the western French–Spanish Basque ones, as revealed by *F*_ST_ estimates and isolation by distance and clustering analyses. Some of these particular colonies might be insular (England), but are commonly located near the edge of *R. ferrumequinum* distribution range. Contrasting levels of genetic diversity between insular and continental populations are common in animals (Frankham, 1995, 1995, 1996) and have already been observed between England and the continent in several bat species such as *R. ferrumequinum* (Rossiter et al., 2000), *Myotis bechsteinii* (Wright et al., 2018), *R. hipposideros* (Dool et al., 2013), *Plecotus austriacus* (Razgour et al., 2013), and *Eptesicus serotinus* (Moussy et al., 2015). The contemporary isolation of colonies from UK with the continent might have maintained higher levels of genetic drift, as shown by the low *N*_e_ estimates, which reinforces their vulnerability (Newman & Pilson, 1997). These colonies may face stochastic reduction of genetic diversity that could limit their evolutionary potential, in particular in the face of environmental changes.

More surprisingly, the northern French colony at Montreuil‐sur‐Mer (“MON”) exhibited genetic patterns that were similar to those observed in the English colonies (lower level of genetic diversity, smaller effective population size, and higher levels of genetic differentiation compared to the other western French–Spanish Basque colonies). This colony was also the only one that exhibited a high proportion of strongly related individuals. Overall, these results suggested that this colony experienced strong genetic drift, due to a small effective population size and limited gene flow with other western French–Spanish Basque colonies. This situation could be explained by a lack of favorable habitat surrounding the colony, leading to limited dispersal and reduced extracolony mating. It could also be explained by the location of the colony at the northern limit of *R. ferrumequinum* distribution range (central‐margin hypothesis, Eckert et al., 2008). Indeed, we observed asymmetric gene flow from the core to the edge of the western French–Spanish Basque population. We also detected a decrease of effective population size and genetic diversity and an increase of genetic differentiation while moving further away from the core (source–sink functioning). Finer population genetics and landscape analyses in north‐eastern France and in Belgium should assess whether this pattern can be extrapolated to all colonies located within this northern limit of *R. ferrumequinum* distribution range or whether it is very specific to this colony.

The last particular situation emphasized in this study concerned Tunisia. The level of genetic diversity detected in this colony was close to the levels observed in the western French–Spanish Basque colonies, but private allele richness was higher. Moreover, this Tunisian colony exhibited a signature of demographic expansion, although we have to be cautious with this result as the confidence interval of the estimated time since the expansion started was extremely large. Larger and denser sampling of colonies of *R. ferrumequinum* from North Africa would be necessary to assess whether it might apply to all colonies located on this southern part of the Mediterranean Sea.

### 4.4 Implications for conservation

In this study, we have shown that connectivity, genetic diversity levels, and effective population size are high and homogeneous in the western French–Spanish Basque population, when excluding the northern French colony “MON.” Therefore, the French Poitou‐Charentes region does not need to be considered as a management unit by itself. We rather recommend considering the large population as a unique management unit. The development of new partnerships or the reinforcement of existing ones between NGOs from different neighboring countries (Spain, France) and French administrative regions are needed to improve the knowledge and conservation of this (and other) bat species in France. This large population could be resilient to local disturbance because of its strong interconnection between colonies. However, we cannot exclude that some particular colonies within this population might be vulnerable. It is therefore still important to pursue colony surveys at local scales and also to standardize monitoring procedures at the national and international scale (Battersby, 2010). Our sampling scheme did not enable us to identify the eastern boundaries of this population or to test for large geographical barriers to gene flow. It would require more sampling in Eastern France and in neighboring countries (e.g., Belgium, Germany, Luxembourg, and Switzerland). In the future, this delineation and inference of bat population demography might be of particular importance as *R. ferrumequinum* experienced severe declines there, so that some populations might be at higher risk of extinction and deserve special management attention (see Ransome & Hutson, 2000).

We have also shown in this study that peripheral colonies are genetically poorer than those at the core range, because of genetic drift, low gene flow, and small effective population size. Thus, these colonies are more vulnerable to extinction and deserve particular management efforts. Interestingly, these colonies located at the edge of the species range are genetically divergent and may harbor some genetic and phenotypic variability that could be important for adaptation to global changes (Lesica & Allendorf, 1995). For example, these colonies may play a key role in the face of climate change by facilitating species range shift northward (Rebelo, Tarroso, & Jones, 2010).

Lastly, our results advocate for paying particular attention to mating territories and to movement pathways that enable extracolony mating. Conservation programs should include the identification and protection of mating site and their associated habitats, and the maintenance of connected and structured semi‐open habitats (i.e., mosaic landscapes of broadleaf woodland and grassland connected with tree lines) that are needed for bats to complete their yearly life cycle. Further collaborations between NGOs and academics are required to evaluate these relationships between landscapes, movements at different scales, mating, and genetic mixing, through the development of joint ecological and genetic approaches.

## CONFLICT OF INTEREST

None declared.

## AUTHOR CONTRIBUTIONS

O.T., D.P., N.C., M.L., and J.‐B.P have conceived the study; M.L., J.‐B.P, and D.P. have organized the fieldwork; M.L., J.‐B.P, O.F.‐C, J.D., O.T., A.L., N.C., I.G., F.M., S.P., and D.P. have helped in collecting the biological material; O.T. and A.L. have conducted the DNA analyses; O.T., R.L., and S.P have analyzed the data; O.T., N.C., and D.P. have drafted much of the manuscript. All authors read, criticized, and approved the final manuscript.

## Data Availability

Microsatellite genotypes for this study are available at: https://doi.org/10.5061/dryad.r44t5dk.
